# Seventh BMC ecology image competition: the winning images

**DOI:** 10.1186/s12898-020-00310-w

**Published:** 2020-08-07

**Authors:** Alison L. Cuff, Michel Baguette, Simon Blanchet, Luke M. Jacobus, Dominique Mazzi, Josef Settele

**Affiliations:** 1 BMC, Berlin, Germany; 2 Muséum National d’Histoire Naturelle, Paris, France; 3 Laboratoire d’Ecologie Expérimentale du CNRS à Moulis, Saint-Girons, France; 4 Indiana University-Purdue University Columbus (IUPUS), Columbus, Indiana USA; 5 Agroscope, Wädenswil, Switzerland; 6 Helmholtz-Centre for Environmental Research – UFZ, Leipzig, Germany

## Abstract

The seventh BMC Ecology competition attracted entries from talented ecologists from around the world. Together, they showcase the beauty and diversity of life on our planet as well as providing an insight into the biological interactions found in nature. This editorial celebrates the winning images as selected by the Editor of *BMC Ecology* and senior members of the journal’s editorial board. Enjoy!

## Editorial

The 7th edition of our image competition has, in common with previous years [1–6], produced a stunning collection of images. Our entrants have demonstrated their talents in presenting their research, using photography to highlight their work.

As always, our *BMC Ecology* Section Editors lent their expertise to judge the many entrants to the competition, selecting the best images from their area of research as well as the overall winner. This ensures that the winning images are selected not only on the basis of quality and beauty but for the scientific story behind them.

As with all previous competitions, we are delighted with the excellent standard and diversity of the images submitted. The judging proved to be a welcome distraction from the coronavirus pandemic for all the editors involved.

We hope that you enjoy looking through all our winning and highly commended entries.

## Winning images

### Overall winner

**Fig. 1:**
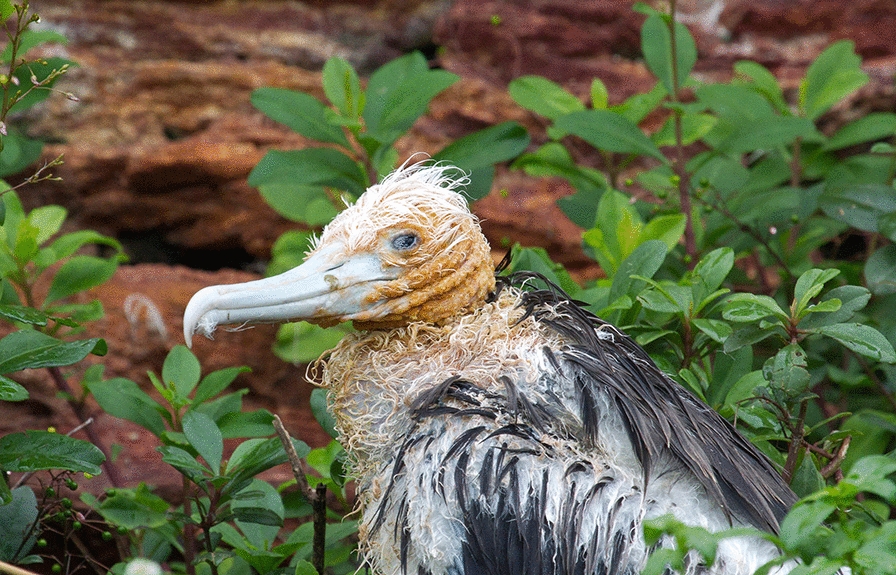
Overall winner: Viruses are a significant ecological concern because they are responsible for a variety of pathological effects in wildlife. This picture shows a chick of Magnificent frigatebird (Fregata magnificens) with clear signs of a viral disease that gives low chances of recovery. Attribution David Costantini

Our overall winning image of a magnificent frigatebird (Fregata magnificens) chick, was taken by David Costantini from the Muséum National d’Histoire Naturelle, Paris, France (Fig. 1). This large seabird is found over the tropical and subtropical waters off America, as well as the Cape Verde islands and Galápagos Islands. The chick is suffering from a viral infection from which it isn’t likely to recover. David Costantini says “The photograph was taken in French Guiana, where viral outbreaks affect annually a population of frigatebirds. An ongoing research project is trying to figure out the causes and consequences of this disease and to find out solutions for the conservation of the local frigatebird population, which is one of the most important of South America”.

Our Conservation Ecology and Biodiversity Research section editors both recommended the entry. Luke Jacobus states that “This image is timely as—at this writing—the world focuses on the interaction between a virus and a metapopulation of animals, which happens to be comprised of humans. It is my hope that lessons from ecology and conservation biology will bear fruit and help us to rise, meet and mitigate this challenge and to improve responses in the future”. Josef Settele said: “I found this picture important because of the message behind it. While everybody nowadays is aware of viruses and their potential impact on humans, we must not forget that there are numerous diseases out there which are affecting many different species—and that these diseases are part of nature and our environment. Although natural, it may be us humans who often disturb the balance which may lead to pandemics for humans and others species alike.”

### Runner up

**Fig. 2:**
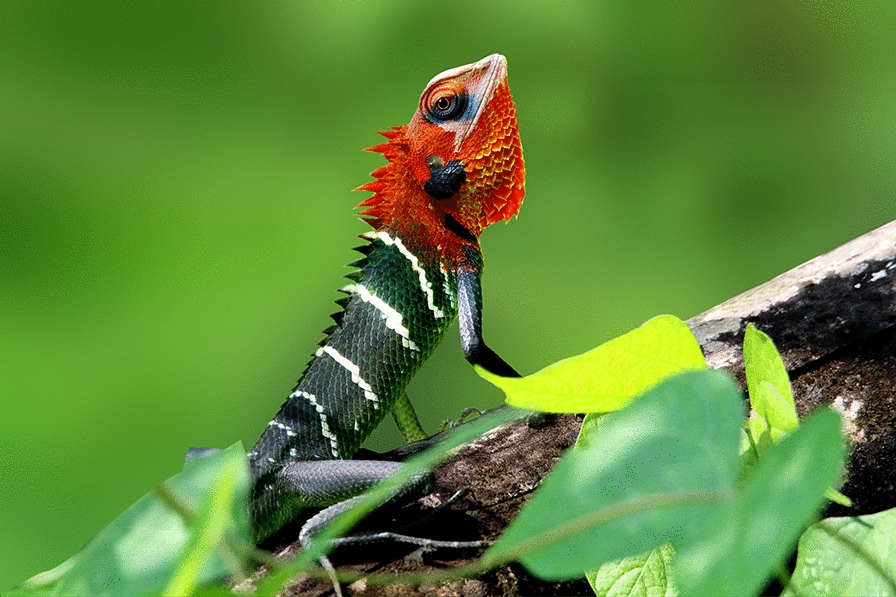
The forest green lizard (Calotes calotes) is large among the lizard species measuring 50–65 cm, from its head to tail. This lizard, as its name suggests, has a bright green dorsal color with 5–6 cream or deep green transverse stripes. These stripes continue to its tail. During the breeding season, the male lizard’s head and throat will turn bright red, while the underside of its body turns pale green. Attribution S S Suresh

Our runner up this year provides quite a visual contrast to our overall winner, showcasing the vibrant colors of a forest green lizard (Fig. 2).

This reptile lives in forests in India and Sri Lanka and is one of the larger species of iguanian lizard. The photographer, Dr S S Suresh from the Ibri Regional Referral Hospital in Oman, describes how they came across their beautiful subject: “It was my routine travel home to Kerala and I had planned an outing to fulfill my desire and passion to capture some wildlife images, soon after my obligatory visits to meet my family and friends. Final arrangements were set and the rest was a feeling of excitement with a shade of anxiety. The climate was not on my side, as it was a wet, gloomy season. As usual, packed with positivity, I set out on a rainy November morning accompanied by two forest rangers. It was a mix of fun-filled and bizarre feeling as we trekked those long and hardy 10 km, covered with dense evergreen forests of Kerala. Brimming with euphoria, my utmost sole aim was to capture a tiger or at least a wild dog through the lenses. I was not disheartened as the tigers remained invisible but was delighted to spot a few species of birds such as the drongos, small minivets and a few wetland birds as we explored the sanctuary. However, luck was on my side, as my friends spotted a flamboyant pair of lizards in their breeding plumage. I was elated and that made my day”.

Josef Settele singles the image out for a special mention saying: “I simply found this picture so beautiful and in contrast to the winner “Sick” that I thought it would nicely pair up with my winner selection and especially show the spectrum of how species can appear to the observer.”

### Behavioral and physiological ecology

**Fig. 3:**
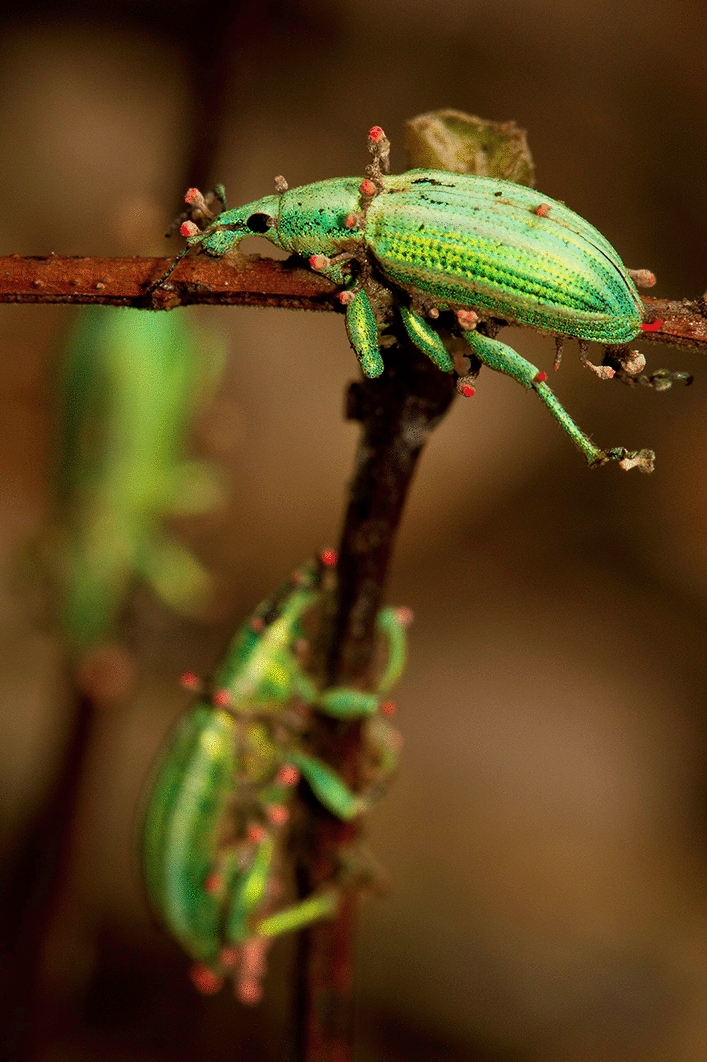
Weevils infected by the “zombie fungus” Cordyceps. Attribution Damien Esquerré

Our winner in this category is entitled “Zombie fungus” and was taken by Damien Esquerré from the Australian National University (Fig. 3). He says that the photograph shows “some species of weevil (Curculionidae), that got infected and killed by Cordyceps, known as the “zombie fungus”, that infects specific species of insects, controls their behaviour and ultimately kills them. The fungus then comes out of its body to release spores to continue its cycle”.

Section editor Dominique Mazzi says “it perfectly captures the helplessness of the weevil affected by the fungus, which before killing its host takes over its behaviour, likely in order to enhance the fungus’ transmission. The off-focus fellow adds to the dark atmosphere of the image, contrasted against its shrill colours, and appears just as doomed, meekly waiting its turn to surrender its fate to the fungus’ benefits”.

### Community, population and macroecology

**Fig. 4:**
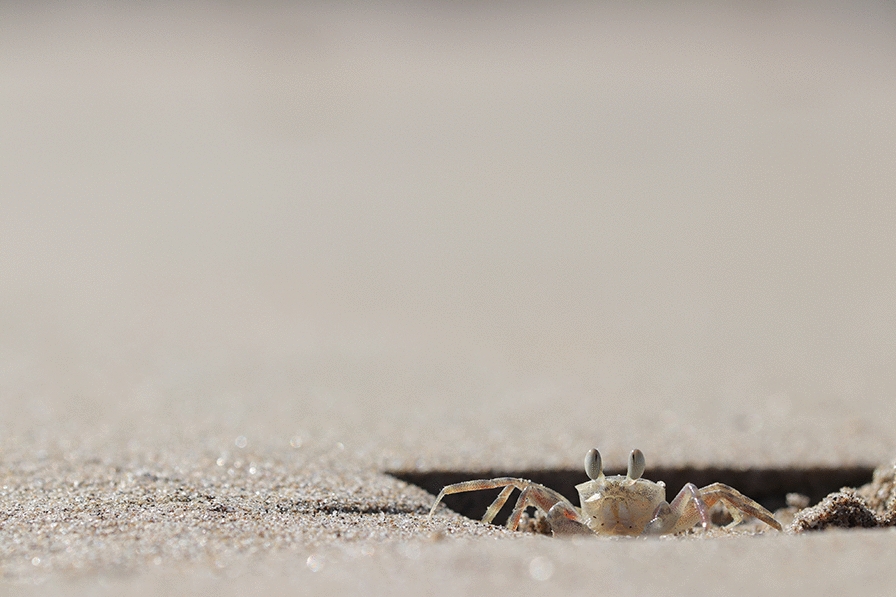
A ghost crab hiding in a human footprint on a beach in China. Attribution HaoYun Zhuang

The winning image in this category is that of a ghost crab, taken by HaoYun Zhuang from Fuzhou University (Fig. 4). These crabs are commonly found on the beaches of tropical and subtropical regions across the world, including in China, where this picture was taken. Their name is a reflection of both their nocturnal nature and their pale coloring. HaoYun Zhuang mentions in their description that this “ghost crab is hiding in the shadow of human’s footprint” and that they “dig caves in the beach meanwhile they can move very fast to escape from the predators like egrets”.

### Conservation ecology and biodiversity research

**Fig. 5:**
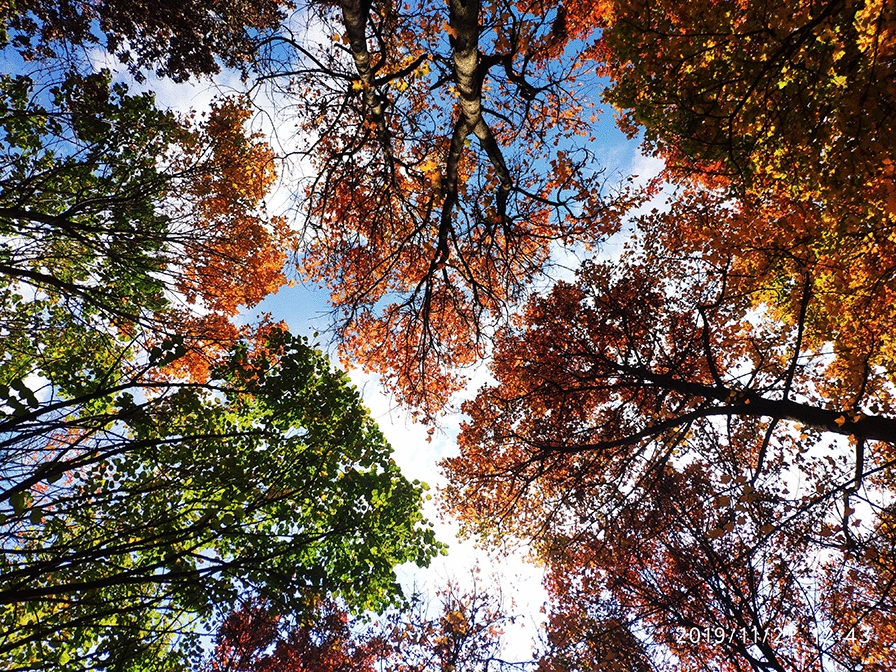
A beautiful demonstration of the “crown shyness” effect amongst different species of tree. Attribution Zu-Chang Xu

Our winner in this category was an entry by Zu-Chang Xu from the Chinese Academy of Sciences. It demonstrates a phenomenon called “crown shyness”, whereby the crowns of fully stocked trees do not touch each other, resulting in a canopy with channel-like gaps. It is most often seen among trees of the same species, but can happen among groups of trees of different species as well. And indeed, this latter scenario is what is being shown in the image (see Fig. 5) as Zu-Chang Xu explains “ In the forest system, different species’ response to the climate is not synchronized, and there is a “ Crown shyness” effect between the canopies of the trees, thus forming a special forest pattern. This picture combines these two elements to form a wonderful landscape”.

Judge Luke Jacobus states that the photograph “illustrates different aspects of biodiversity, including species richness and relative abundance. The various colors are an accessible and immediately recognizable proxy for more technical measures of forest diversity.”

### Landscape ecology and ecosystems

**Fig. 6:**
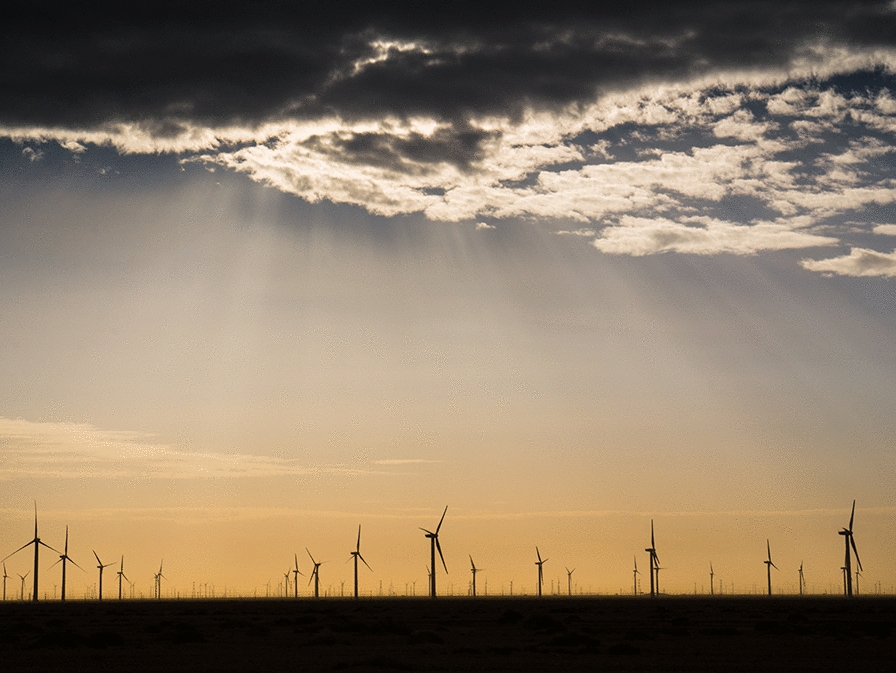
A wind farm in China’s Gobi Desert. Attribution Kang Xu

Kang Xu, who is from the College of Life Sciences, Zhejiang University is our winner in this category, submitting a stunning image of a wind farm in China’s Gobi Desert (Fig. 6). They state that “China has led the global wind market for ten consecutive years, accounting for 35% of the global installed capacity. Guazhou, which is in Gansu Province and is known as the “World Wind Library”, owns the largest wind farm cohort in the world. Currently, the wind energy generated in Guazhou can reach levels greater than 20 GW, which is roughly equivalent to that of all of Spain (i.e., 23 GW in 2017, ranked fifth in the world after Germany). Our previous study demonstrated that constructing wind turbines in the Gobi Desert is a win–win strategy that both contributes to the growth of desert vegetation with a favourable microclimate and sufficiently utilizes wind power to produce clean energy. During the field study, we took the spectacular photograph of the largest wind farm-desert coupled ecosystem”.

### Editors pick

**Fig. 7:**
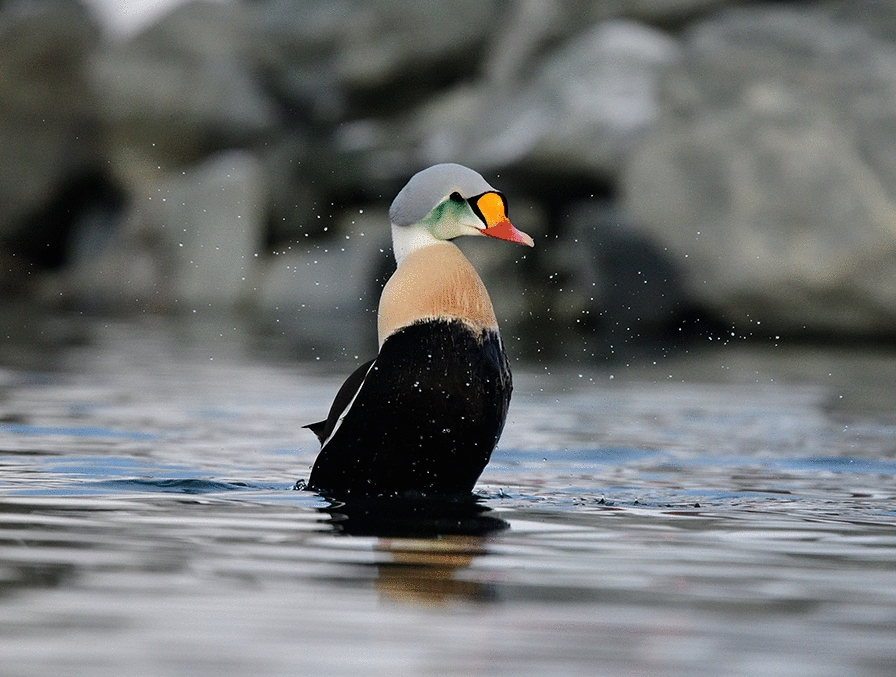
A King eidar duck bathing in some shallow water. Attribution Nayden Chakarov

My choice as the Editor is entitled “The Kings Bath” and it was captured by Nayden Chakarov from Bielefeld University, Germany (Fig. 7).

It shows a large sea duck called the king eider having a splash in the water. I chose it due to the vibrancy of the image of the bird against an almost monochrome background as well as it being in stark contrast to our overall winner of this competition.

Nayden Chakarov tells us more “King eiders breed only in the highest Arctic territories. Along with other eider ducks, kings are known for their extremely insulating feathers. During the long Polar winter, king eiders migrate south and spend most time in the open ocean. Occasionally kings visit the harbours of Nordaustlandet and other relatively “warm” territories, where they eagerly feed and bathe in the shallow waters. Opportunistic breeding leads to strong fluctuations in size and reproductive success of king eider populations, depending on the suitability of environmental conditions. Despite its large range and population size, this northernmost duck species may be severely impacted by rising temperatures in the high Arctic”.

In short, it’s a cheerful photo of a duck having a wash.

## Highly commended

Beyond our winning images we received many other fantastic photos. Our favourites are showcased below.

### Behavioral ecology and interactions with the environment

Some of the strongest entrants for this competition captured elements of an animal’s behavior and how they interact with their environment.

One of my favourites is an image entitled “Fly on the fly” (Additional file 1) taken by Bing Lin from Princetown University. The photographer captures beautifully that moment where a young gelada monkey has had enough of the fly that has been buzzing in her ear, attempting to get at the insect as it still flies around her.

Meercats usually bring with them the “aww” factor and the image entitled “The art of idleness” (Additional file 2) taken by our overall winner, David Costantini is no exception. In this entry, he takes a photo of a female meerkat taking a rest after foraging for food.

The photo entitled “Green Mantis” (Additional file 3) was taken by one of our winners, Damien Esquerré. It captures an interaction between an Australian green mantis laying her eggs and a spider; the arthropod jumps at the mantis and was probably quite lucky if it didn’t become lunch.

### Conservation ecology and biodiversity

Entrants in this category were also very good this year; here are a few of the highlights.

Edgardo Londoño-Cruz took the image entitled “Evolving” (Additional file 4) while resting from doing some research when a Foureye rockskipper decided to join him. This amazing intertidal fish is found in rocky tidal pools, is able to breathe air and can live up to 4 h in damp environments.

The image “Shyness” (Additional file 5) is of a *Sphaeropteris cooperi*, an ornamental tree fern found in Australia that can grow to up to 15 m in height. Its fronds are quite delicate and can be killed by frosts. The frond captured here by Heyu Lin from the University of Melbourne is tightly coiled against the early spring air.

Diatoms are unicellular algae commonly found in oceans and soils. They produce 20% of the oxygen on the planet. Some occur as solitary cells but Luca Santangeli’s competition entry, titled “Diatom chain” (Additional file 6) is an confocal image of a colony taken at the EMBL light microscope facility in Heidelberg.

### Stunning landscape

We conclude by showcasing another beautiful picture taken by the overall winner of the competition, David Costantini of a lightning storm in the Kalahari Desert (Additional file 7).

## Conclusions

We were delighted at the variety and quality of the images submitted to the 7th BMC Ecology image competition. Thank you to everyone who submitted an entry and congratulations to the winners! We hope that our readers enjoy their work as much as we have enjoyed judging them. We look forward to next year’s competition!

## Supplementary information

**Additional file 1.** I took this picture while I was conducting scientific field research on the behavioral ecology of gelada monkeys (Theropithecus gelada) in Guassa, Ethiopia.Geladas are Old-World primates endemic to Ethiopia. They are the only grass-eating primates left in the world and are the last remaining species of their genus still alive today. As such, they are a critical study species to consider in attempting to unravel the many secrets of primate evolution. This photo shows a fly bothering this adolescent female, and I took this shot right as she became fed up with the fly buzzing in her ear, a moment I could empathize well with. An adolescent female gelada monkey bites and claws at a bothersome fly in mid-flight. Attribution Bing Lin (Princeton University, USA).

**Additional file 2.** Meerkats (Suricata suricatta) spend a large amount of their active time foraging. This female meerkat, radio-tracked by scientists for hours under the sunlight of Kalahari, decided to take a break and enjoy some minutes of idleness. I was right there to capture this moment using a Canon EOS 7D (400 mm, f/13, 1/640 s). Minor adjustments (e.g.,sharpening) were applied. Attribution David Costantini (Muséum National d'Histoire Naturelle, Paris, France).

**Additional file 3.** This photo has a special story. I was photographing this Australian green mantis (Orthodera ministralis) which was laying eggs, when suddenly a spider jumped from the ground on it and engaged on a two second battle with the mantis eventually throwing the spider off to the ground. I was so happy to manage to snap a photo and capture this incredible moment. Taken in Round Hill, NSW, Australia. Attribution Damien Esquerré (Research School of Biology, The Australian National University).

**Additional file 4.** This picture was taken in March 2008 during a biodiversity research campaign to the rocky shores of Chocó (Colombian Pacific). I was "resting" searching for small creatures in an intertidal pool when I found this little one (Dialommus macrocephalus) crawling out of the water to take a "sun-bath". Working with invertebrates, I was amazed at how this little fellow was so relaxed in an "unnatural" environment. Attribution Edgardo Londoño-Cruz (Universidad del Valle).

**Additional file 5.** This picture was taken at Dandenong Ranges National Park in Australia, showing the unexpanded frond of an Australian tree fern (Sphaeropteris cooperi),which is native to Australia. While the frond seems very shy and tightly curled in the early spring, it can rapidly unfurl and grow up to 5 m long afterward. Ferns not only provide habitats and shelters for animals but also act as a bioindicator for the health of the ecosystem, as they filter and purify the air of various toxins. Attribution Heyu Lin(School of Earth Sciences, The University of Melbourne, Australia).

**Additional file 6.** Twenty percent of the oxygen we breathe is produced in the ocean by minute algae known as diatoms. Some are solitary, some form chain-like colonies, like these specimens (Thalassiosira), collected near the coasts of the Antarctic peninsula by the schooner of the TARA Oceans project for plankton research. This confocal image, taken at the EMBL light microscopy facility, shows the diatoms’ cell wall (cyan), chloroplasts (red), DNA (blue), membranes and organelles (green). Attribution Luca Santangeli (Arendt Lab, EMBL Heidelberg).

**Additional file 7.** Heat waves in the Kalahari are often followed by impressive lightning storms. A real sense of electricity permeates the environment. I got this shot using a Canon EOS 7D (18 mm, f/3.5, 1/8000 s). Minor adjustments (e.g., sharpening) were applied. Attribution David Costantini (Muséum National d'Histoire Naturelle, Paris, France).
