# Supplementary figures and images for: Seventh BMC ecology image competition: the winning images
Source: BMC Ecol. 2020 Aug 7;20:42. doi: 10.1186/s12898-020-00310-w (PMC7410526; doi:10.1186/s12898-020-00310-w)

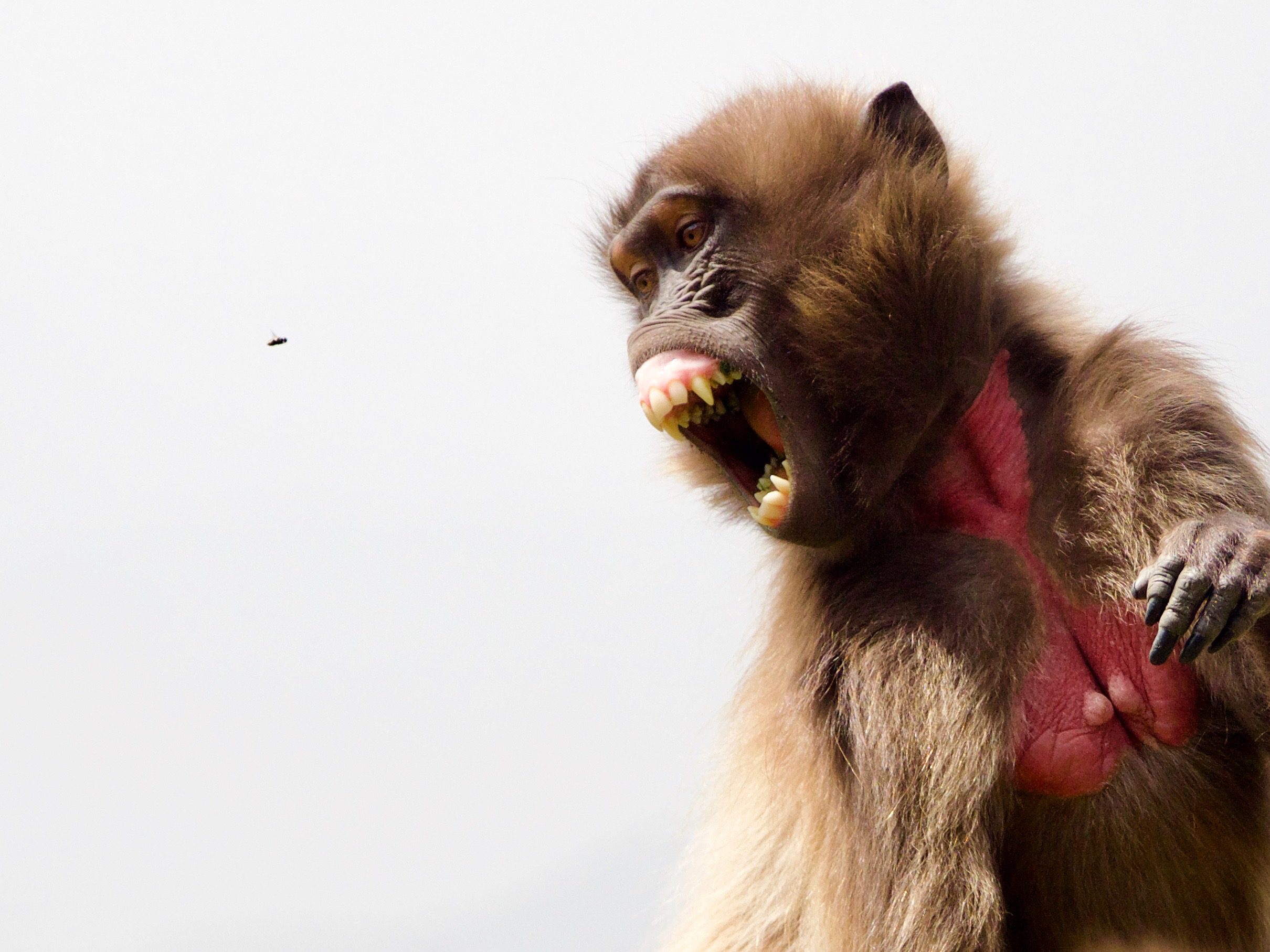

Supplement: Supplementary file 1 — Additional file 1. I took this picture while I was conducting scientific field research on the behavioral ecology of gelada monkeys (Theropithecus gelada) in Guassa, Ethiopia.Geladas are Old-World primates endemic to Ethiopia. They are the only grass-eating primates left in the world and are the last remaining species of their genus still alive today. As such, they are a critical study species to consider in attempting to unravel the many secrets of primate evolution. This photo shows a fly bothering this adolescent female, and I took this shot right as she became fed up with the fly buzzing in her ear, a moment I could empathize well with. An adolescent female gelada monkey bites and claws at a bothersome fly in mid-flight. Attribution Bing Lin (Princeton University, USA). [file 12898_2020_310_MOESM1_ESM.jpg]

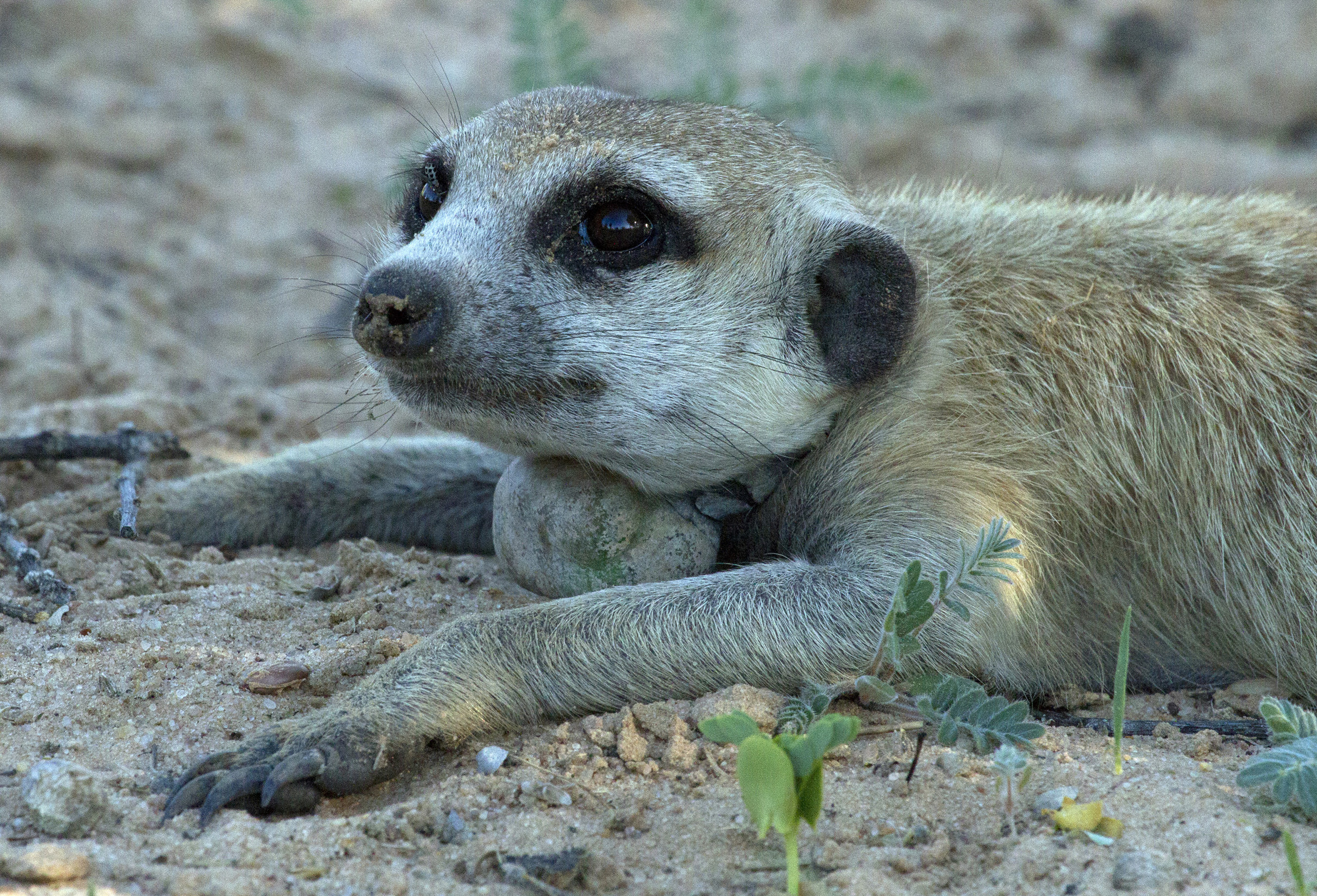

Supplement: Supplementary file 2 — Additional file 2. Meerkats (Suricata suricatta) spend a large amount of their active time foraging. This female meerkat, radio-tracked by scientists for hours under the sunlight of Kalahari, decided to take a break and enjoy some minutes of idleness. I was right there to capture this moment using a Canon EOS 7D (400 mm, f/13, 1/640 s). Minor adjustments (e.g.,sharpening) were applied. Attribution David Costantini (Muséum National d'Histoire Naturelle, Paris, France). [file 12898_2020_310_MOESM2_ESM.jpg]

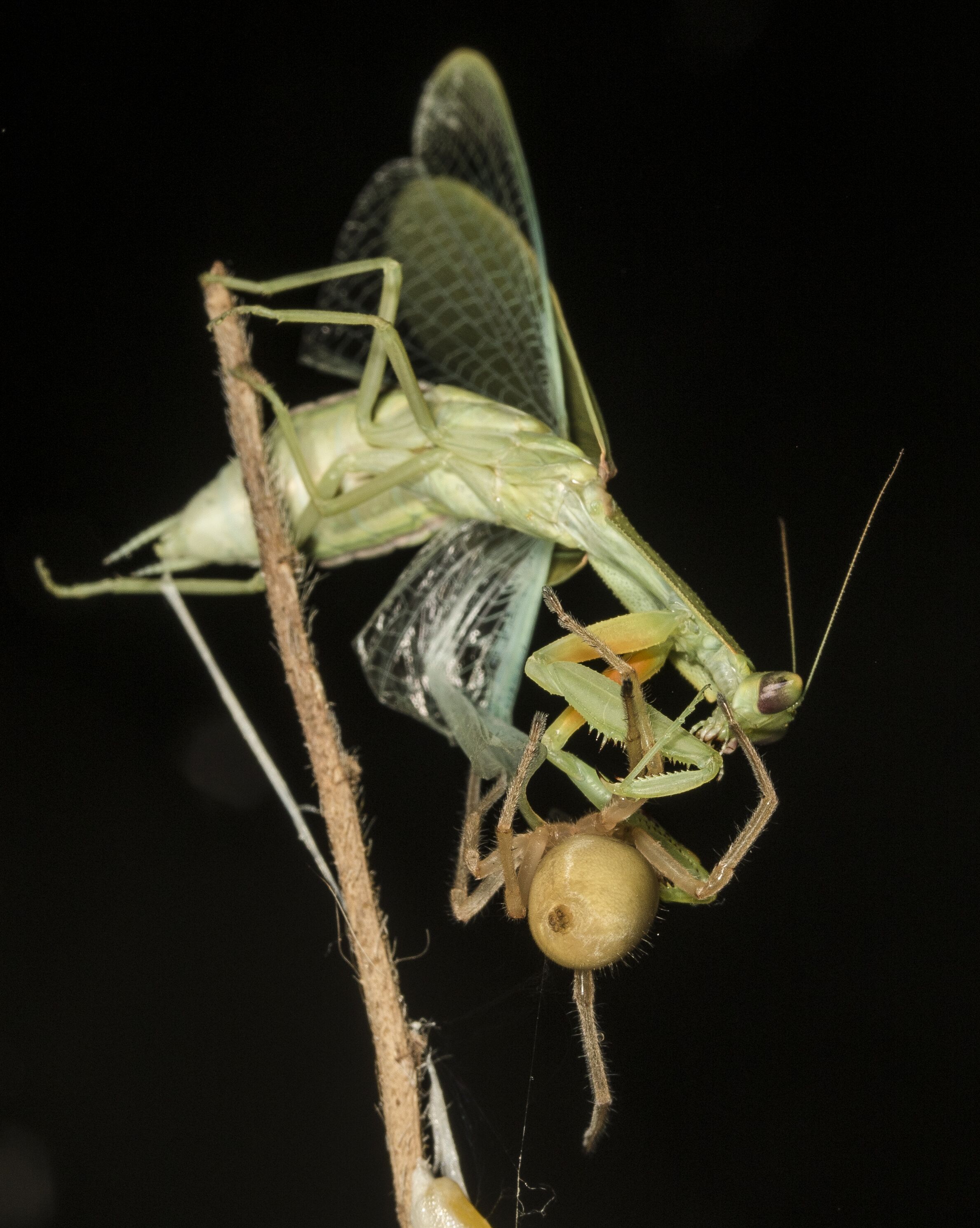

Supplement: Supplementary file 3 — Additional file 3. This photo has a special story. I was photographing this Australian green mantis (Orthodera ministralis) which was laying eggs, when suddenly a spider jumped from the ground on it and engaged on a two second battle with the mantis eventually throwing the spider off to the ground. I was so happy to manage to snap a photo and capture this incredible moment. Taken in Round Hill, NSW, Australia. Attribution Damien Esquerré (Research School of Biology, The Australian National University). [file 12898_2020_310_MOESM3_ESM.jpeg]

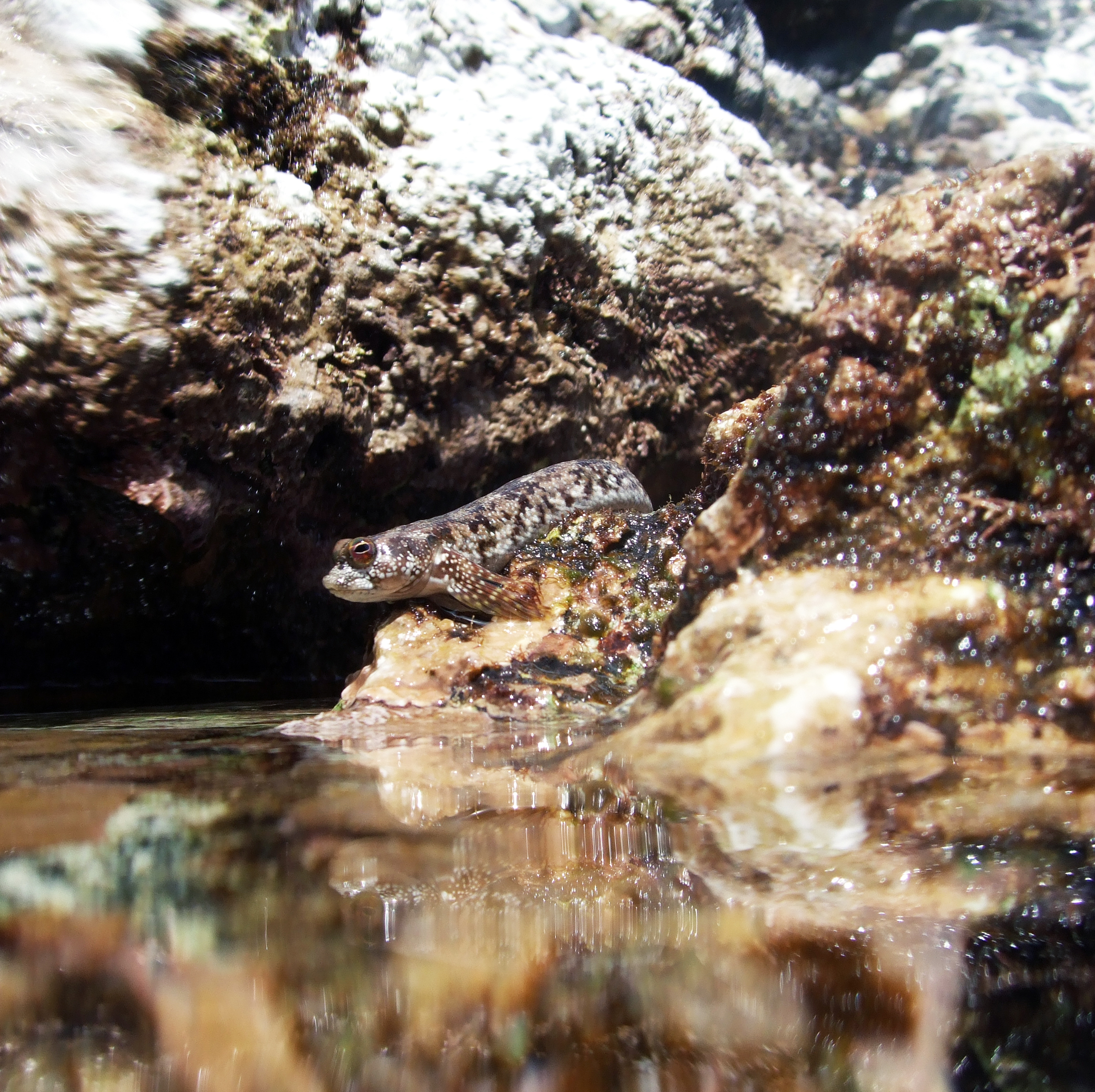

Supplement: Supplementary file 4 — Additional file 4. This picture was taken in March 2008 during a biodiversity research campaign to the rocky shores of Chocó (Colombian Pacific). I was "resting" searching for small creatures in an intertidal pool when I found this little one (Dialommus macrocephalus) crawling out of the water to take a "sun-bath". Working with invertebrates, I was amazed at how this little fellow was so relaxed in an "unnatural" environment. Attribution Edgardo Londoño-Cruz (Universidad del Valle). [file 12898_2020_310_MOESM4_ESM.jpg]

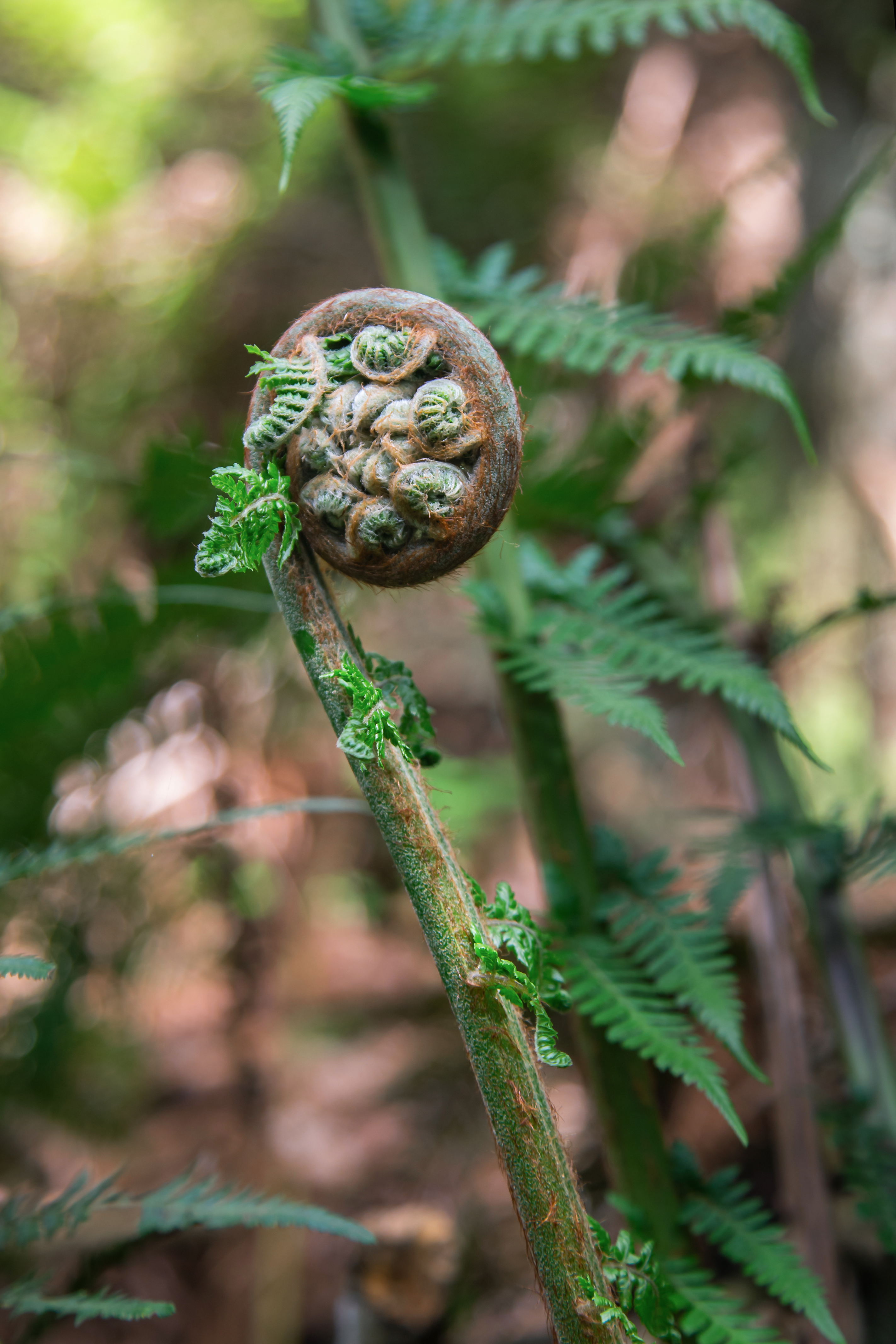

Supplement: Supplementary file 5 — Additional file 5. This picture was taken at Dandenong Ranges National Park in Australia, showing the unexpanded frond of an Australian tree fern (Sphaeropteris cooperi),which is native to Australia. While the frond seems very shy and tightly curled in the early spring, it can rapidly unfurl and grow up to 5 m long afterward. Ferns not only provide habitats and shelters for animals but also act as a bioindicator for the health of the ecosystem, as they filter and purify the air of various toxins. Attribution Heyu Lin(School of Earth Sciences, The University of Melbourne, Australia). [file 12898_2020_310_MOESM5_ESM.jpg]

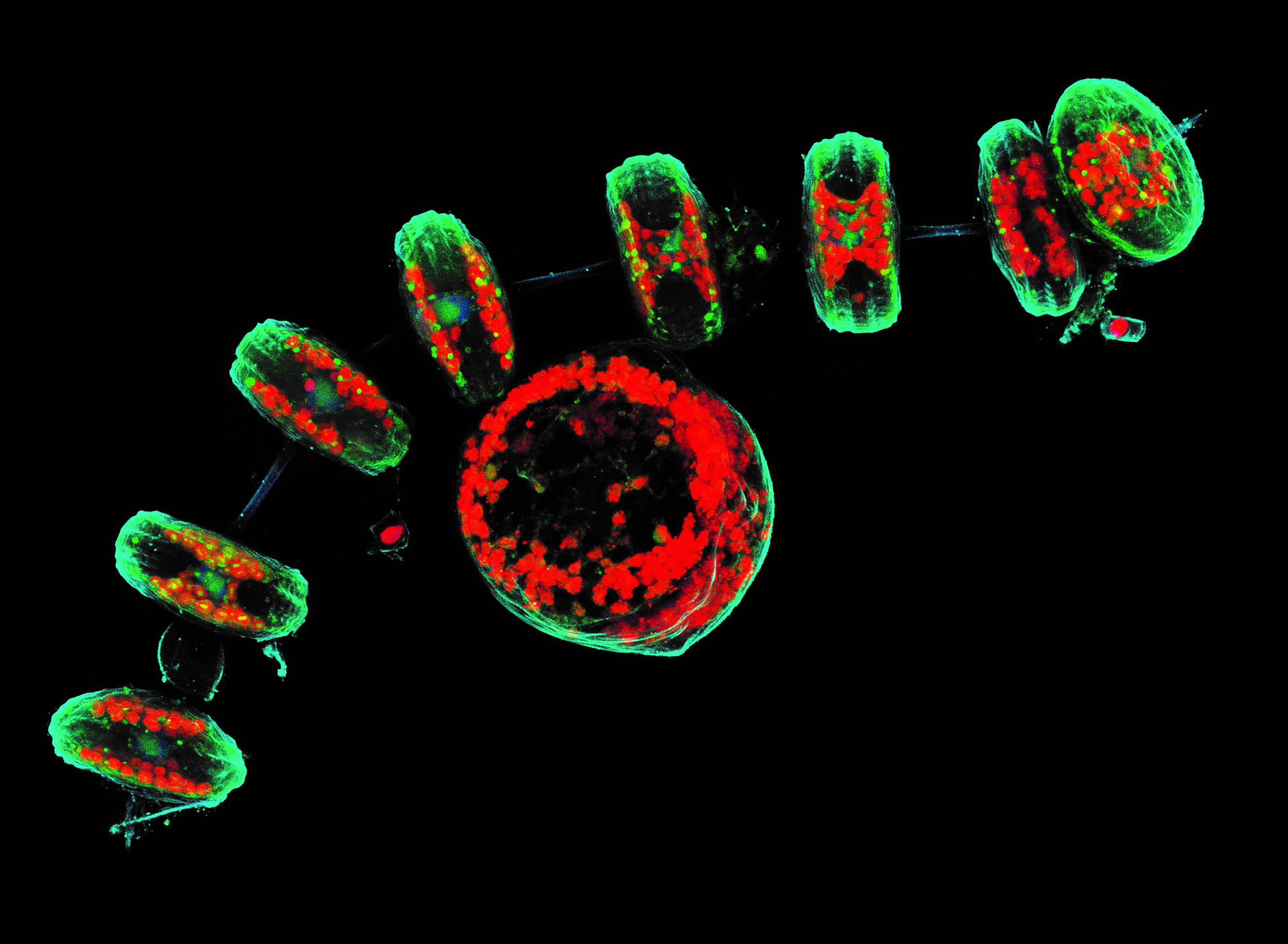

Supplement: Supplementary file 6 — Additional file 6. Twenty percent of the oxygen we breathe is produced in the ocean by minute algae known as diatoms. Some are solitary, some form chain-like colonies, like these specimens (Thalassiosira), collected near the coasts of the Antarctic peninsula by the schooner of the TARA Oceans project for plankton research. This confocal image, taken at the EMBL light microscopy facility, shows the diatoms’ cell wall (cyan), chloroplasts (red), DNA (blue), membranes and organelles (green). Attribution Luca Santangeli (Arendt Lab, EMBL Heidelberg). [file 12898_2020_310_MOESM6_ESM.tif]

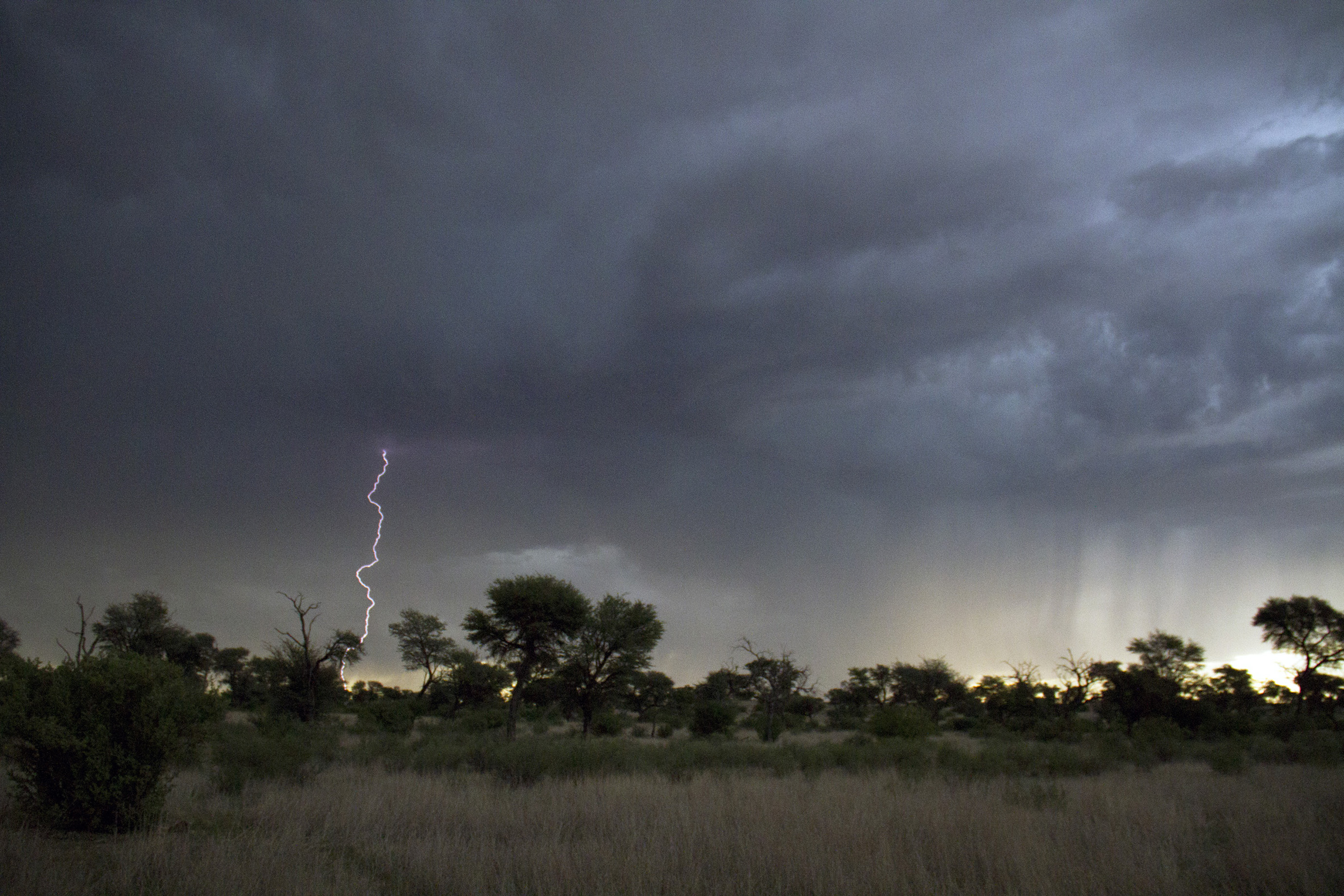

Supplement: Supplementary file 7 — Additional file 7. Heat waves in the Kalahari are often followed by impressive lightning storms. A real sense of electricity permeates the environment. I got this shot using a Canon EOS 7D (18 mm, f/3.5, 1/8000 s). Minor adjustments (e.g., sharpening) were applied. Attribution David Costantini (Muséum National d'Histoire Naturelle, Paris, France). [file 12898_2020_310_MOESM7_ESM.jpg]
